# The Mitochondrial Disulfide Relay System: Roles in Oxidative Protein Folding and Beyond

**DOI:** 10.1155/2013/742923

**Published:** 2013-11-14

**Authors:** Manuel Fischer, Jan Riemer

**Affiliations:** Cellular Biochemistry, University of Kaiserslautern, Erwin-Schrödinger Straße 13, 67663 Kaiserslautern, Germany

## Abstract

Disulfide bond formation drives protein import of most proteins of the mitochondrial intermembrane space (IMS). The main components of this disulfide relay machinery are the oxidoreductase Mia40 and the sulfhydryl oxidase Erv1/ALR. Their precise functions have been elucidated in molecular detail for the yeast and human enzymes *in vitro* and in intact cells. However, we still lack knowledge on how Mia40 and Erv1/ALR impact cellular and organism physiology and whether they have functions beyond their role in disulfide bond formation. Here we summarize the principles of oxidation-dependent protein import mediated by the mitochondrial disulfide relay. We proceed by discussing recently described functions of Mia40 in the hypoxia response and of ALR in influencing mitochondrial morphology and its importance for tissue development and embryogenesis. We also include a discussion of the still mysterious function of Erv1/ALR in liver regeneration.

## 1. Introduction

Because almost all proteins in eukaryotic cells are synthesized by cytosolic ribosomes, protein translocation across membranes is critical for organelle biogenesis. The invention of organelle-specific targeting systems in the cytosol was instrumental to facilitate correct translocation events and to avoid mistargeting. These pathways are usually complemented by machineries in the organelle lumen which provide driving force and ensure directionality. For example, in the endoplasmic reticulum (ER) and the mitochondrial matrix members of the Hsp70 family of chaperones bind incoming substrates and thereby prevent their backsliding (ratchet-like mechanism) [1]. A similar mechanism is employed for protein import into the mitochondrial intermembrane space (IMS). Here formation of inter- and intramolecular disulfide bonds by the essential mitochondrial disulfide relay is critical for translocation across the mitochondrial outer membrane [2–6]. In this review, we will discuss the disulfide relay and its components, compare and contrast the machineries in yeast and human cells, and discuss additional potentially nonoxidative functions of disulfide relay components in human cells.

## 2. Substrates of the Mitochondrial Disulfide Relay

**Figure 1:**
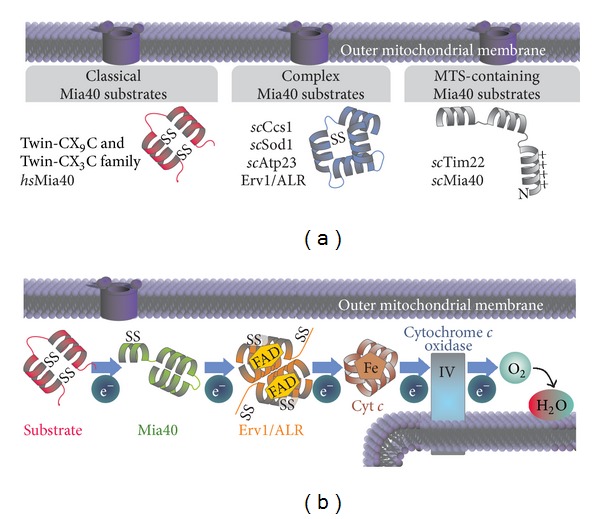
Substrates and general outline of the mitochondrial disulfide relay. (a) Mia40 substrates can be classified into three groups: (1) members of the twin-CX_9_C and twin-CX_3_C family, respectively. Members of both families rely on four cysteines localized within two *α*-helices for proper import. (2) The proteins *sc*Ccs1, *sc*Sod1, *sc*Atp23, and Erv1/ALR form a second group of substrates with more complex folds and disulfide patterns. So far no common signal for the interaction with Mia40 has been identified in these proteins. (3) The two MTS-containing Mia40 substrates Tim22 and *sc*Mia40 are imported in a membrane potential-dependent manner and require Mia40 for proper folding only. (b) General outline of oxidative folding in the IMS. During substrate oxidation electrons are transferred from the substrate to Mia40. To reoxidize Mia40 electrons are transferred further via ALR to cytochrome *c* (Cyt *c*) and then to cytochrome *c* oxidase. Molecular oxygen (O_2_) is used as final electron acceptor to finally yield water (H_2_O).

Most proteins that are imported into mitochondria contain either a mitochondrial targeting signal (MTS) or internal targeting sequences [4, 7, 8]. They are thereby targeted into the mitochondrial matrix or to the two mitochondrial membranes. In contrast, only few of the precursors of IMS proteins carry the so-called bipartite presequences consisting of an MTS and a hydrophobic sorting region [8, 9]. The import of the majority of soluble IMS proteins is facilitated by the mitochondrial disulfide relay system in a process that is linked to the oxidative folding of the proteins [3, 10] (Figure 1). Most of the so far identified disulfide relay substrates belong to the families of twin-CX_3_C proteins or twin-CX_9_C proteins (C, cysteine; X, any amino acid) (Figure 1(a)). The members of both families are small proteins with most of them having a size of around 10 kDa. They share a common simple core structure that consists of two antiparallel alpha helices arranged in a helix-loop-helix motif [11]. Each helix contains two cysteines that are separated by either three or nine amino acids for members of the twin-CX_3_C or twin-CX_9_C families, respectively [11–16]. Twin-CX_3_C or twin-CX_9_C proteins fulfill diverse functions within the IMS. They serve as chaperones for newly imported proteins, are involved in metal transfer and insertion during respiratory chain biogenesis, or are part of mature respiratory chain complexes [13, 17–22]. In human and yeast cells exist a total of five proteins that belong to the twin-CX_3_C family. Conversely, the twin-CX_9_C family appears to be significantly larger in mammalian cells, and in addition numerous proteins exist that do not adhere exactly to the nine amino acid-wide spacing (and instead have, e.g., CX_8_C or CX_10_C motifs). So far more than 30 twin-CX_9_C family members have been identified in human cells, and some of them have been confirmed to be disulfide relay substrates [23, 24].

In addition to twin-CX_3_C and twin-CX_9_C proteins several more complex substrates exist that rely on the mitochondrial disulfide relay for oxidation (Figure 1(a)). In yeast the import of the dually localized copper chaperone for superoxide dismutase 1 (Ccs1) and in part also that of superoxide dismutase 1 (Sod1) depends on the mitochondrial disulfide relay [25–27]. Likewise, import and oxidation of the sulfhydryl oxidase Erv1 which itself is part of the mitochondrial disulfide relay (see below) are driven by the disulfide relay system [28]. Further substrates are the mitochondrial protease Atp23 and the inner membrane protein Tim22 [29, 30]. The latter protein contains a bipartite presequence and thus requires oxidation only for folding but not for mitochondrial import. Because so far a systematic identification of interaction partners and substrates of the mitochondrial disulfide relay system is lacking in yeast and mammalian cells, we do not know how large the group of disulfide-containing IMS proteins is. It is likely that it will be significantly larger than previously anticipated as the recently solved partial IMS proteome contains numerous proteins that are dually localized between cytosol and IMS, but lack MTS, and might therefore be disulfide relay substrates [31].

## 3. The Mechanism of Oxidation-Dependent Protein Import by the Mitochondrial Disulfide Relay System

**Figure 2:**
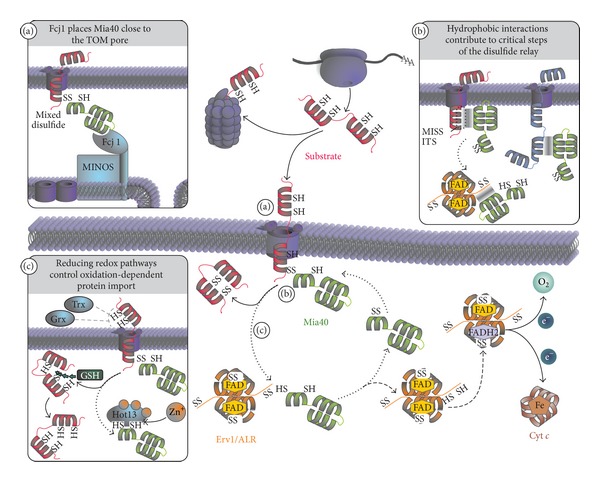
The mitochondrial disulfide relay system facilitates protein import and folding into the IMS. Classical substrates of the twin-CX_3_C and twin-CX_9_C families are translated on cytosolic ribosomes. In part, these proteins are degraded by the proteasome, while the majority becomes posttranslationally imported into the IMS through the TOM pore. Noncovalent and covalent interactions between Mia40 and the substrate are necessary for translocation and oxidative folding of the substrate. Immediately after the first cysteine of the substrate translocates a mixed disulfide between Mia40 and substrate is formed. The substrate becomes oxidized by resolving the mixed disulfide complex. Reduced Mia40 is then reoxidized by the flexible N-terminal domain of one subunit of Erv1/ALR, allowing another round of substrate oxidation. Within the Erv1/ALR homodimer electrons are transferred from the N-terminal cysteines of one subunit to the C-terminal cysteines of the other subunit from where they are shuttled to the prosthetic FAD molecule. Erv1/ALR then passes electrons onto cytochrome *c*—and further to cytochrome *c* oxidase and oxygen yielding H_2_O as product. Alternatively, electrons can be transferred from the FAD directly onto oxygen thus forming H_2_O_2_. (a) The MINOS complex is important for the organization of the IMS in yeast. Both the arrangement of the cristae and the close proximity of the TOM and TIM pore are mediated by MINOS. Fcj1 binds to the MINOS complex and also interacts with Mia40, thereby placing Mia40 close to the TOM pore. (b) Hydrophobic interactions between the hydrophobic groove of Mia40 and twin-CX_3_C and twin-CX_9_C proteins are necessary for substrate recognition by Mia40. The same hydrophobic patch on Mia40 also mediates its interaction with the N-terminal domain of ALR. Moreover, the hydrophobic groove of Mia40 also equips the protein with a holdase-like function that can serve in importing cysteine-less substrates. (c) Several redox control pathways facilitate efficient oxidative import and folding of substrates. In the cytosol substrate cysteines are maintained in their reduced state mainly by thioredoxins (Trx) and glutaredoxins (Grx) in yeast and human cells, respectively. During Mia40-dependent oxidation reduced glutathione (GSH) exhibits a proofreading function by reducing wrongly oxidized substrates and resolving trapped intermediates of substrate and Mia40. Upon becoming reduced, the cysteines of Mia40 are prone to bind zinc ions, thereby interfering with reoxidation. The zinc chelating protein Hot13 keeps Mia40 zinc free.

All proteins of the IMS are synthesized by cytosolic ribosomes [32] (Figure 2). However, only very few of them contain a classical MTS or internal targeting signals that guide them to mitochondria. Instead, most IMS proteins contain conserved cysteine patterns or other still ill-defined motifs that are recognized by the IMS-localized mitochondrial disulfide relay but likely also by so far not identified cytosolic factors [4, 10]. Such factors could ensure targeting of disulfide relay substrates to mitochondria for posttranslational import and maintain them in an import-competent unfolded state. In addition, disulfide relay substrates have to be kept in a reduced state in the cytosol. This is facilitated by cytosolic glutaredoxins in human cells and the thioredoxin system in yeast [33, 34] and potentially by the presence of zinc ions that can complex reduced cysteines [35, 36]. In yeast, the amounts of import-competent substrates are also controlled by the cytosolic proteasome system that degrades substantial amounts of newly synthesized disulfide relay system substrates before they can be imported [37]. At present it remains unclear whether such a degradation pathway is also found in mammalian cells and under which conditions it may serve in adapting amounts of imported IMS proteins.

Translocation of IMS proteins takes place across the translocase of the outer membrane (TOM). Upon exposure of a recognition motif termed MISS or ITS (for mitochondrial intermembrane space sorting and IMS-targeting signal, resp.) disulfide relay substrates are recognized by the protein Mia40 (for mitochondrial IMS import and assembly; in mammalian cells also CHCHD4) [14, 38], which thereby serves both as import receptor and chaperone and oxidoreductase [23, 29, 39] (Figure 2, insets (a) and (b)). Mia40 consists of a structural helix-loop-helix motif with two stabilizing disulfide bonds that form hydrophobic substrate recognition and binding groove and a redox-active CPC motif that is positioned in a flexible helix which hovers over the substrate binding site [40, 41]. Yeast and human Mia40 share high homology, except for an N-terminal extension in yeast Mia40 that contains a bipartite presequence which is lacking in the human protein. Human Mia40 appears in two different splice variants (CHCHD4.1 and CHCHD4.2) [42, 43]. They are completely identical except for the very N-terminal part of the protein. The isoform 1 does contain an additional cysteine at position four; however, whether the isoforms exhibit different functionality or substrate specificity is not known. Like for ALR the import and folding of human Mia40 depend on the disulfide relay system [44]. In contrast, yeast Mia40 requires the disulfide relay system only for oxidative folding [45]. In yeast, Mia40 is positioned close to the *trans*-side of the TOM complex by its interaction with Fcj1 (for formation of cristae junction; in human cells mitofilin) [46] (Figure 2, inset (a)). Fcj1 is part of the MINOS complex which organizes the topology of the cristae in the inner mitochondrial membrane [46–50]. 

MISS/ITS motifs have been well defined for classical twin-CX_3_C and twin-CX_9_C proteins but their nature has to be clarified for the growing class of nonclassical substrates like Atp23. It contains hydrophobic residues, and in most cases, a single cysteine residue, that are positioned on the same side of an alpha helix [14, 38] (Figure 2, inset (b)). After recognition of the MISS/ITS signal by the hydrophobic binding groove of Mia40, the thiolate anion of a cysteine in the substrate performs a nucleophilic attack on the oxidized CPC motif of Mia40 which results in the formation of an intermolecular disulfide bond [2, 39]. This disulfide bond together with the hydrophobic interactions between substrate and Mia40 prevents the backsliding of the incompletely translocated substrate into the cytosol, thus coupling import to oxidative protein folding [51]. Consequently, mutation of critical cysteines in Mia40 substrates also results in very low amounts of these substrates in the IMS [2]. This indicates that hydrophobic interactions with Mia40 might be sufficient to drive IMS import at least of some proteins that neither contain classical MTS nor cysteines to interact with Mia40. Furthermore, Mia40 might also contribute to protein folding as it is capable of stabilizing cysteine-free unfolded proteins and prevents their aggregation [29] (Figure 2, insert (b)).

The intermolecular disulfide bond between substrate and Mia40 is resolved by another nucleophilic attack of a thiolate anion in the substrate leaving an oxidized substrate molecule and a reduced Mia40 molecule [39]. Thus, for this import mechanism to work Mia40 substrates have to contain at least two cysteines. For the introduction of more than one disulfide bond, multiple oxidized Mia40 molecules or molecular oxygen are necessary. It has been suggested that Mia40 can act more efficiently in the introduction of multiple disulfide bonds by forming a ternary complex with its substrate and the essential protein Erv1 (in mammalian cells augmenter of liver regeneration (ALR), growth factor erv1-like (Gfer1), hepatopoietin or *hs*Erv1) [52, 53]. The very rapid introduction of disulfide bonds after the formation of the initial intermolecular disulfide bond is supported by the fact that *in vivo* no semioxidized intermediates of substrate proteins can be observed [23].

The sulfhydryl oxidase Erv1/ALR acts to reoxidize the CPC motif in Mia40 [39]. It is a homodimeric protein in which each subunit consists of two domains [54]. The first—N-terminal domain—contains a redox-active CXXC motif and serves as a “shuttle arm” that mediates the electron transfer from Mia40 to a CXXC motif in the C-terminal core domain of Erv1/ALR [39, 55]. To this end, this mainly unstructured domain interacts with the substrate-binding groove of Mia40 [56, 57]. Consequently, overexpression of Erv1/ALR in intact cells delays oxidative protein folding and IMS import because the N-terminal arm blocks substrate binding to Mia40 [23]. Both Mia40 and Erv1/ALR are perfectly adapted to this critical interaction of shuttle arm and hydrophobic groove. In a heterologous yeast system human ALR or human Mia40 when expressed individually could to a large part complement their yeast counterparts. However, only if human Mia40 and human ALR were concomitantly used to substitute the respective yeast proteins full complementation was ensured [44]. After Mia40 reoxidation the shuttle arm of Erv1/ALR swings over to the core domain of the second subunit of Erv1/ALR (intersubunit electron transfer) and becomes reoxidized by the core CXXC motif [55]. This core CXXC motif is reoxidized by the redox cofactor of Erv1/ALR-flavin adenine dinucleotide (FAD) by the formation of a charge-transfer complex [58]. The FAD is held in place by the very compact four-helix bundle structure of Erv1/ALR [54, 59, 60]. The dimer of Erv1/ALR is stabilized by hydrophobic interactions and in mammalian cells additionally also by disulfide bonds [39, 61]. Finally, the reduced FAD cofactor is reoxidized by either transferring electrons directly onto molecular oxygen which gives rise to the production of hydrogen peroxide or alternatively by transferring electrons to cytochrome *c* [39, 62, 63]. To which extent both pathways are utilized in intact cells remains unclear although *in vitro* cytochrome *c* appears to be the preferred electron acceptor [39, 58, 62, 64, 65]. At least in yeast cells Erv1 can transfer electrons also onto an anaerobic electron acceptor [66]. The identity of this acceptor and whether it is conserved in mammalian cells remains unclear.

In addition to Mia40 and Erv1/ALR further factors modulate oxidative folding in the IMS—the protein helper of Tim protein (Hot13, in mammalian cells RCHY1) and the local glutathione pool (Figure 2, insert (c)). Hot13 is a cysteine-rich protein that is capable of chelating zinc ions [67]. Although low amounts of zinc ions can facilitate mitochondrial import *in vitro*, too high amounts hamper substrate oxidation and Mia40 reoxidation by binding to reduced cysteines [36, 67, 68]. It has thus been proposed that Hot13 keeps the CPC motif of Mia40 in a zinc-free state thereby accelerating oxidation-dependent protein import. *In vitro* substrate oxidation appears to yield side products with nonnative disulfides or substrates that are trapped in their mixed disulfide complex with Mia40 [39]. Formation of these products is avoided by the presence of reduced glutathione. Also in intact cells reduced glutathione seems to be beneficial for oxidation-dependent protein import: on the one hand by contributing to the reduced redox state of Mia40 substrates in the mammalian cytosol, and on the other hand by accelerating oxidative protein folding by a still unresolved mechanism [23]. Like zinc ions glutathione might be a two-edged sword. The IMS glutathione pool in yeast and mammalian cells has been measured to be as reducing as the one in the cytosol [23, 69]. The IMS glutathione redox potential is thereby in the range of the redox potential of Mia40 substrates, raising the question of how these substrates can be oxidized and maintained in an oxidized state [35, 65, 69–73]. Although this point has not been addressed experimentally, it is likely that the thermodynamically feasible reduction of Mia40 substrates is kinetically prevented, for example, hampering the equilibration between protein thiols and glutathione. In principle glutathione can affect IMS proteins *in vivo* as the CPC motif of Mia40 is affected by glutathione in intact cells [23, 69]. Consequently, Mia40 is maintained in a partially reduced state in yeast cells [69]. The reduced part of molecules might well be involved in either isomerisation or reduction reactions like oxidoreductases in other systems that facilitate oxidative protein folding. However, such a novel role of Mia40 has not been shown.

**Figure 3:**
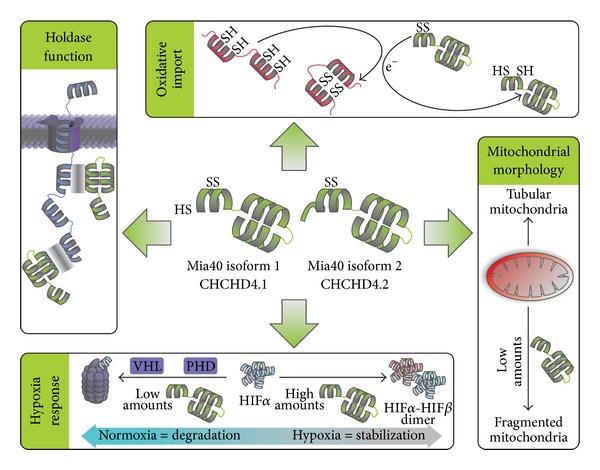
Functions of human Mia40. The protein exists in two different isoforms. Differences in function between the isoforms are not known. *Oxidative Import*. Mia40 is the main receptor for import and oxidation of twin-CX_9_C and twin-CX_3_C proteins in the IMS. *Holdase Function*. Mia40 shields hydrophobic patches on proteins imported into the IMS, thus allowing proper folding of these proteins. *Mitochondrial Morphology*. Reduced amounts of Mia40 lead to increased mitochondrial fission and the formation of a more fragmented mitochondrial network. *Hypoxia Response*. Under normoxic conditions the protein HIF1*α* is constantly degraded. This degradation is mediated by hydroxylation by prolyl hydroxylase domain enzymes (PHD) and ubiquitination by the Hippel-Lindau E3 ligase (VHL). Upon hypoxia oxygen as substrate of PHD is missing resulting in impaired HIF1*α* degradation and increased stability. If not degraded HIF1*α* can dimerize with constitutively expressed HIF1*β* and induce the hypoxia response. Silencing Mia40 using RNAi prevents stabilization of HIF1*α* under hypoxic conditions. In contrast increased amounts of Mia40 as can also be found in certain tumors result in increased stabilization of HIF1*α*.

**Figure 4:**
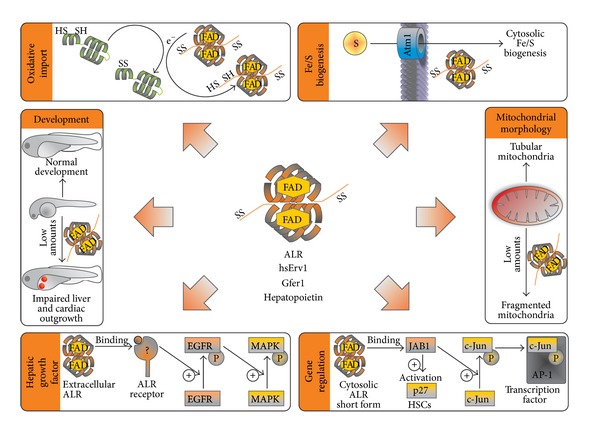
Functions of Erv1/ALR. *Oxidative Import*. Erv1/ALR reoxidizes Mia40. *Fe/S Biogenesis*. In yeast Erv1/ALR is required for the biogenesis of cytosolic Fe/S proteins but not for mitochondrial Fe/S proteins. *Development*. Erv1/ALR is expressed during development. Knockdown or chemical inhibition of Erv1/ALR leads to impaired development of organs such as liver and hamper cardiac outgrowth. *Mitochondrial Morphology*. Erv1/ALR is important for mitochondrial morphology in undifferentiated cells. Knockdown of Erv1/ALR in mouse embryonic stem cells leads to mitochondrial fragmentation and increased levels of Drp1 (dynamin-related protein 1). *Hepatic Growth Factor*. Extracellular Erv1/ALR increases regenerative capacities of liver tissue. Extracellular Erv1/ALR can bind to a so far unknown receptor. Upon binding tyrosine-phosphorylation of the epidermal growth factor receptor (EGFR) is enhanced which promotes phosphorylation and activation of mitogen activated protein kinase (MAPK). *Gene Regulation*. A truncated cytosolic form of Erv1/ALR can bind to Jun activating binding protein 1 (JAB1). Binding to JAB1 increases JAB1-mediated phosphorylation of c-Jun. Upon phosphorylation c-Jun can form a complex with AP-1 complex (activator protein 1). In hematopoietic stem cells (HSC) sequestering of JAB1 by ALR prevents binding of JAB1 to p27(kip).

Besides their function in oxidative protein folding in mitochondria Mia40 and Erv1/ALR also function in potentially unrelated (nonmitochondrial) pathways. Mia40 was shown to be critical for mitochondrial dynamics and the hypoxia response [43] (Figure 3). For Erv1/ALR, a plethora of different cellular and physiological functions were described (Figure 4). Erv1/ALR influences fusion and fission processes of mitochondria [74–77]; it is important for the development of certain organs during embryogenesis [78, 79] and functions as mitogen to enhance regenerative capacities of liver tissue [80–82]. These functions will be discussed in the following.

## 4. Physiological Impact of Mia40 and Erv1/ALR

### 4.1. A Function of Mia40 in Hypoxia

Mia40 is not only necessary for proper assembly of the respiratory chain but is also involved in the stabilization of hypoxia inducing factor 1*α* (HIF1*α*) [43, 74] (Figure 3). In the presence of high amounts of oxygen HIF1*α* is continuously degraded by the proteasome after hydroxylation by oxygen-dependent prolyl hydroxylase domain (PHD) enzymes and subsequent ubiquitinylation by the E3 ligase VHL (von Hippel-Lindau) [83–85]. Under low oxygen conditions PHDs lack oxygen and fail to completely hydroxylate HIF1*α*. Moreover, reactive oxygen species take part in the stabilization process by further inhibiting PHD [86, 87]. The stabilization of HIF1*α* by low oxygen concentrations can be mimicked by incubating cells with iron chelators as PHD activity depends on an iron cofactor [88–90].

The modulation of Mia40 levels affects HIF1*α* stabilization at low oxygen concentration but not by treatment with iron chelators [43]. Upon depletion of Mia40 using siRNA-mediated knockdown HIF1*α* failed to accumulate under low oxygen conditions, while Mia40 overexpression enhanced HIF-1*α* stabilization under hypoxic conditions. Since the hypoxia response is critical for tumor growth Mia40 depletion effectively inhibited tumor growth and angiogenesis *in vivo *[43]. In line with these findings in human cancer, increased Mia40 expression was found to correlate with the signature of hypoxia gene expression [43]. Whether the described effect of Mia40 on the stabilization of HIF1*α* arises from a direct interaction or is indirectly mediated by an impaired respiratory chain remains unclear and is an exciting question for future research.

### 4.2. Physiological Functions of Erv1/ALR—in Mitochondria and the Cytosol?

Human patients with a homozygous mutation in Erv1/ALR exhibit respiratory-chain deficiency, myopathy, congenital cataract, sensorineural hearing loss, and delayed development [59, 91] (Figure 4). In zebrafish the formation of heart and liver is impaired upon chemical inhibition or silencing of Erv1/ALR [78, 79]. In addition, chemical inhibition of Erv1/ALR induces apoptosis in human embryonic stem cells [79]. Likewise, silencing of Erv1/ALR in mouse embryonic stem cells results in caspase-induced apoptosis as well as in excessive fragmentation of mitochondria and elimination of damaged mitochondria through mitophagy [76, 77]. Taken together these data underline the importance of Erv1/ALR for mitochondrial functionality especially during development. 

Most of those physiological effects of Erv1/ALR likely derive directly or indirectly from its role in the mitochondrial disulfide relay (Figures 2 and 4). However, they might also be linked to a role in the biogenesis of cytosolic iron sulfur proteins which has been described for yeast Erv1 [92]. Moreover, they may derive from a so far unappreciated function of Erv1/ALR in the cytosol where overexpressed and tagged Erv1/ALR could be detected in some studies [92]. Unfortunately, overexpression of IMS proteins without bipartite MTS frequently results in mislocalization to the cytosol [23]. It thus remains unclear whether endogenous Erv1/ALR also is dually localized. Besides full length Erv1/ALR a shorter isoform consisting only of the C-terminal core domain has been described to exist in the cytosol and nucleus of mammalian cells and to be secreted as a growth factor [93, 94]. The existence of this isoform has been confirmed by immunoblotting of human cell lysate against endogenous ALR although specificity controls using siRNA-mediated knockdown were lacking in these studies. 

### 4.3. A Role for Erv1/ALR in Liver Regeneration

Erv1/ALR has been described to enhance the regenerative capacities of liver tissue [95–97]. For this role of Erv1/ALR two different mechanisms were proposed. In one model Erv1/ALR acts extracellularly as mitogen [81]. Several studies describe the regeneration-enhancing abilities of Erv1/ALR on damaged liver tissue after application of the purified C-terminal domain of human or rat Erv1/ALR [80]. The C-terminal domain can be cross-linked to a 60 kDa protein which is probably located at the cellular surface [81]. The putative Erv1/ALR receptor does not interact with other mitogenic factors such as epidermal growth factor (EGF), transforming growth factor *α* (TGF-*α*), or insulin [81]. Binding of Erv1/ALR to its receptor triggers EGF-receptor phosphorylation which then results in activation of the mitogen-activated protein kinase (MAPK) signaling cascade [82]. 

In a second model cytosolic Erv1/ALR acts independently of the MAPK pathway. Cytosolic Erv1/ALR thereby interacts with Jun-activating domain-binding protein 1 (JAB1) which promotes phosphorylation of c-Jun and therefore formation of the c-Jun/activator protein-1 (AP-1) transcription factor complex [98]. C-Jun is part of the cytosolic COP9 signalosome that has also been shown to interact with Erv1/ALR [99]. The interaction between Erv1/ALR and JAB1 depends on the presence of the CXXC motif in the C-terminal core domain of Erv1/ALR because mutation of the motif to CXXS prevented phosphorylation of c-Jun [100]. The studies addressing the cytosolic function of Erv1/ALR were all either performed *in vitro* using recombinant proteins or by overexpressing Erv1/ALR. As already stated above overexpression of IMS proteins leads to cytosolic or nuclear mislocalization [23]. This is especially true for Erv1/ALR which becomes imported and folded more slowly than classical substrates of the disulfide relay. 

The interaction of Erv1/ALR with JAB1 is not limited to liver cells. Knockdown of Erv1/ALR in hematopoietic stem cells leads to an increased inhibition of the cyclin-dependent kinase inhibitor p27(kip) by JAB1 while overexpression of ALR leads to a decreased inhibition of p27(kip), probably because JAB1 is sequestered by ALR [101]. Furthermore, it was shown that the quiescence promoting properties of ALR in HSC are dependent on Camk4 (Ca^2+^/calmodulin-dependent protein kinase 4). HSCs isolated from Camk4^−/−^ mice possessed reduced levels of ALR and p27(kip) and were deficient in proliferation, which could be restored by ectopic expression of ALR [102].

A complementing explanation for the enhancement of liver regeneration is that Erv1/ALR treatment decreases cytotoxicity of natural killer cells and decreases IFN-*γ* (interferon-gamma) levels [103, 104]. Alternatively, it has been proposed that extracellularly administrated Erv1/ALR enhances liver regeneration by inducing anti apoptotic gene expression, thereby improving cell survival [105]. However, this anti apoptotic effect does not seem to be limited only to hepatocytes because in human lymphocytes recombinant Erv1/ALR also inhibited apoptosis [106]. Recently, it was shown that in primary hepatocytes the increased expression and synthesis of ALR after liver damage is regulated by the transcription factor Nrf2 [107]. This indicates that the regenerative abilities of ALR are not only achieved by extracellular treatment of damaged cells but might constitute physiological relevant cellular survival mechanisms.

### 4.4. The Disulfide Relay System—Open Questions

**Figure 5:**
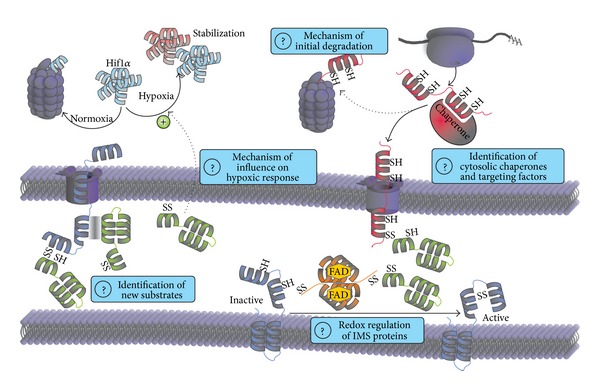
Open questions. *Hypoxia.* Why Mia40 is required for the stabilization of HIF1*α* under hypoxic conditions is unknown. It will be interesting to reveal the underlying mechanism and if the influence of Mia40 is direct or indirect. *New Substrates.* During the last years proteins were found to be dependent on Mia40 that do not share the same motifs as the classical twin-CX_9_C and twin-CX_3_C substrates. They are either dependent on the function of Mia40 as oxidoreductase or holdase. *Initial Degradation and Cytosolic Chaperones/Targeting Factors.* In yeast a portion of IMS proteins is directly degraded after translation. However, it is not known if this takes place in other organisms, how it is regulated, and how it works at the molecular level. Moreover, it remains unclear how proteins after translation are kept import competent and targeted to mitochondria. *Redox Regulation.* The mitochondrial disulfide relay is known to introduce structural disulfide bonds which are required for protein stability. However, it is not known if Mia40 or Erv1/ALR can regulate the activity of IMS proteins by reversible oxidation or reduction.

Mia40 and Erv1/ALR are well characterized regarding their functions as oxidoreductase and sulfhydryl oxidase of the mitochondrial disulfide relay, respectively. Still several open questions remain (Figure 5): first, despite the identification of many human twin-CX_9_C proteins by *in silico* approaches, a concise identification of disulfide relay substrates is still lacking. This becomes especially important because in recent years several proteins with complex structures have been identified as Mia40 substrates. Since these substrates do not adhere to classical cysteine patterns, they cannot be predicted by *in silico* approaches. This might indicate that the substrate range of the disulfide relay is much wider than previously anticipated. It might also include targets for thiol-dependent redox regulation that cycle between oxidized and reduced states and consequently adapt their activities.

Secondly, while we understand oxidative protein folding in the IMS in detail, little is known about the cytosolic processes that take place before translocation across the outer membrane. In previous studies, cytosolic factors were identified which facilitate the import of MTS-containing proteins. However, most disulfide relay substrates lack such targeting information and thus appear like cytosolic proteins. It will therefore be exciting to identify factors that interact with IMS proteins after their translation and guide them to mitochondria or mediate their degradation. 

Thirdly, the role of Mia40 and Erv1/ALR in processes that appear not directly linked to mitochondria such as hypoxia and liver regeneration is still mechanistically ill-defined. It especially remains unclear whether the functions of both proteins in each case are connected to their function in the disulfide relay or if they operate by completely different mechanisms. We think that it will be exciting to establish in detail the molecular mechanisms that underlie these potentially extra-mitochondrial functions and thus link the biochemistry of thiol oxidation with its physiological impact.
